# Leveraging gold nanostars for precision laser interstitial thermal therapy

**DOI:** 10.18632/oncotarget.28592

**Published:** 2024-06-14

**Authors:** Aden P. Haskell-Mendoza, Ethan S. Srinivasan, Tuan Vo-Dinh, Peter E. Fecci

**Keywords:** laser interstitial thermal therapy, gold nanostars, brain tumors, thermal ablation, immunotherapy

Over the past decade, laser interstitial thermal therapy (LITT) has become an important tool for the neurosurgical treatment of a variety of intracranial pathologies, including focal epilepsies, vascular malformations, and central nervous system (CNS) tumors [1]. LITT involves the minimally invasive, stereotactically-guided placement of a laser catheter into a target lesion for subsequent thermal ablation via the delivery of infrared radiation, typically at wavelengths of 980–1064 nm [1, 2]. Following transfer of the patient to a scanner, real-time magnetic resonance (MR) thermometry is employed to track tissue hyperthermal ablation. For patients with primary and metastatic brain tumors who are suboptimal candidates for craniotomy due to clinical status, wound healing concerns, or tumor location, LITT represents a particularly favorable option for reducing tumor burden. However, successful ablation is limited by the (1) inability to precisely sculpt heat to cover or conform to large (typically, ≥3 cm) or irregular lesions, (2) the presence of various intracranial heat sinks, including cerebrospinal fluid (CSF) spaces and blood vessels, and (3) the sensitivity of uninvolved white and gray matter structures to inadvertent thermal damage [1–3].

**Figure 1:**
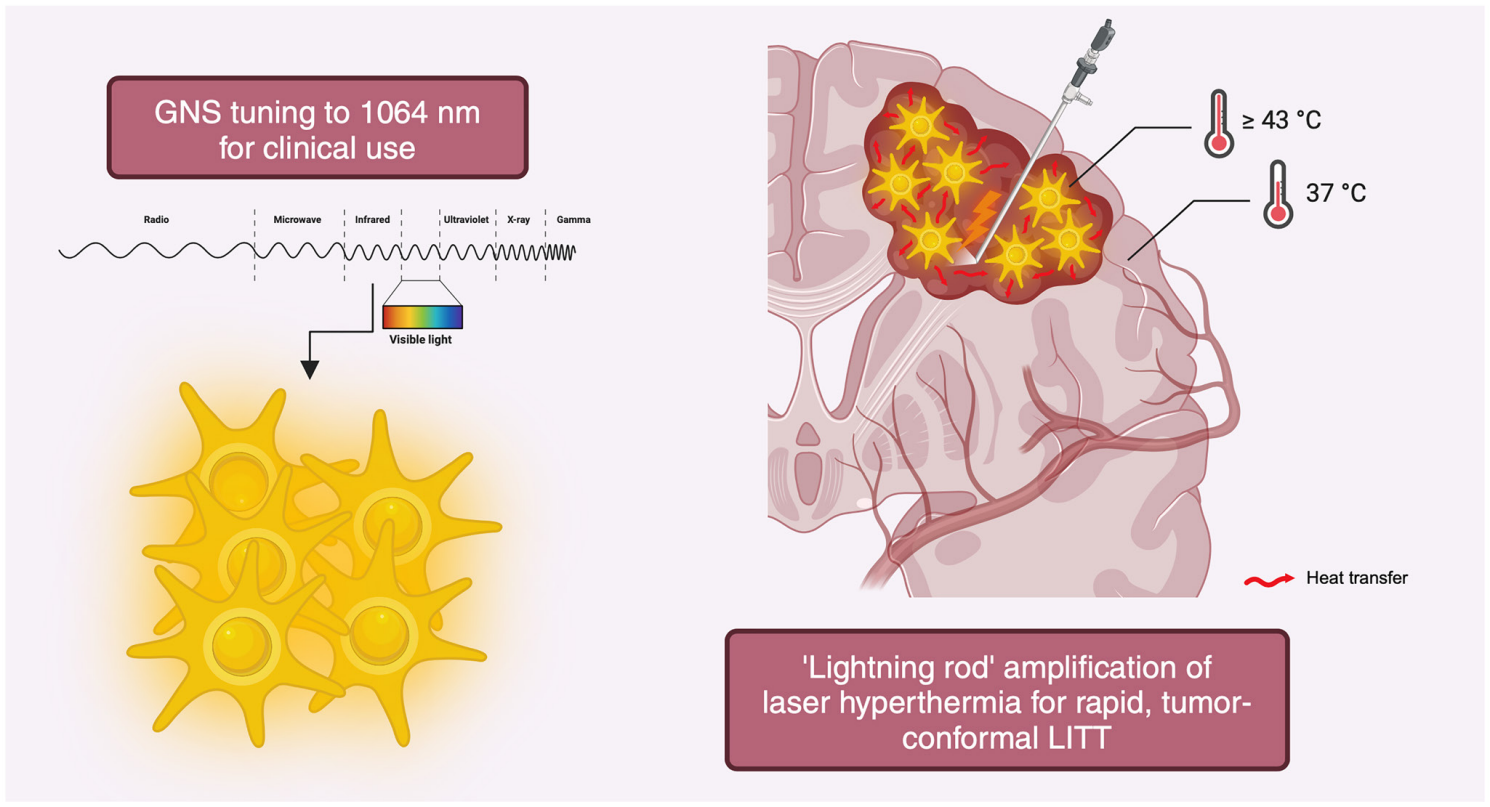
Gold nanostars amplify brain-tumor selective laser interstitial thermal therapy. Schematic representation of GNS tuned to 1064 nm for synergy with clinical LITT (left) with tumor-selective accumulation and amplification of hyperthermia leading to more rapid and conformal ablation that spares adjacent uninvolved tissue (right). GNS not shown to scale. Created with https://www.biorender.com/.

To aid in the performance of more efficient, conformal, and accordingly, safe ablations, we recently developed procedures for the use of gold nanostars (GNS, Figure 1) [2]. GNS can be tuned to specific wavelengths of incident electromagnetic irradiation, which, when delivered to the nanoparticle, cause conduction electrons within the metal to oscillate at an equivalent frequency to that of the delivered light. Such oscillating electrons, termed “surface plasmons”, generate secondary electric fields that result in the accumulation of photothermal energy at curvature points; the star shape of GNS is therefore advantageous, with its many sharp spikes enabling it to act as a ‘lightning rod’ to rapidly and effectively transmit heat via “chain reactions” to immediately surrounding tissue. Notably, GNS accumulate within tumor, but not surrounding uninvolved tissue owing to leaky neovasculature and disruption of the blood-brain barrier (BBB), a phenomenon known as the enhanced permeability and retention (EPR) effect [2]. This phenomenon obviates the need for development of cancer-specific biomolecule tags for homing to tumor *à la* antibody-drug conjugates.

Using a non-toxic, surfactant-free synthesis method, we tuned GNS for maximal photon absorption at the 1064 nm wavelength employed by 2 of the 3 commercially available LITT systems [4]. We then performed *in silico* simulations of photon interactions within tumor and adjacent brain tissue ablated at 1 W power with and without GNS, demonstrating the ability of GNS to focus heat within the tumor volume and limit diffusion to surrounding extratumoral tissue. Leveraging the clinical NeuroBlate LITT system (Monteris Medical, Minnetonka, MN) available at our center, we performed ablations of agarose tumor phantoms with and without GNS under MR thermometry, validating a GNS-mediated increased rate of heating that both expanded and conformed to the distribution of nanostars, even when distributed in irregular (hourglass) or asymmetric (delivered to only half of the tumor phantom) concentrations. Thus, the combination of increased conformality as well as decreased time and energy required for hyperthermia to GNS-bearing tumors indicates the capacity of GNS to protect surrounding eloquent structures from inadvertent thermal damage during LITT.

Motivated by these encouraging results, we next tested the ability of GNS to selectively collect within brain tumors and not surrounding brain tissue. Using a syngeneic murine model of intracranial CT2A glioma, we administered ^124^I radiolabeled GNS via tail vein injection, subjecting the mice to serial PET/CT scans at 10 min, 24 h, and 72 h, demonstrating tumor selectivity at 24 and 72 h timepoints; no ^124^I PET positivity was observed in normal brain tissue or a non-tumor bearing control. In order to verify the EPR effect in both metastatic and primary brain tumors, we performed subsequent two-photon microscopy (TPM) and inductively coupled mass spectrometry (ICP-MS) in both B16F0 melanoma and our CT2A glioma model. Notably, GNS appeared to circulate in both adjacent normal brain and tumor microvasculature at 10 minutes on TPM, while they were exclusively visualized in glioma or metastatic melanoma at 24 and 72 hours. Subsequent ICP-MS at 24 h revealed concentrations of GNS in tumor 22 to 52 times that of normal brain, corroborating the relative specificity of GNS for intracranial tumors without the addition of cancer-type specific targeting ligands.

Having validated the tumor selectivity of GNS, we sought to demonstrate whether GNS could proffer the improved conformality and effective heating we observed in our tissue phantoms *in vivo*. We adapted LITT to mice bearing heterotopic flank CT2A tumors via implantation of a 400 μm laser fiber and thermocouples into the tumor 24 hours after GNS injection. The addition of GNS resulted in higher maximal temperatures achieved when measured at both 2 mm and 4 mm from the tumor core, as well as a faster rate of heating in GNS-injected vs. control mice, replicating the findings of our simulation and phantom studies.

Our study provides multiple levels of evidence demonstrating that GNS serve as lighting rods to amplify the effect of LITT while sparing surrounding normal tissues [2]. Accordingly, cotreatment with LITT and GNS may enable the successful ablation of larger intracranial tumors at lower energy settings. Previously, it was established that GNS are removed from the blood by reticuloendothelial system macrophages and had a favorable toxicity profile 6 months after administration [5]. Notably, an earlier study we performed using extracorporeal near-IR heating of CT2A flank tumors demonstrated synergy between hyperthermia and anti-PD-L1 checkpoint blockade to produce durable tumor regressions [6]. Given its smaller footprint to traditional resection, LITT patients are more rapidly able to resume systemic and/or radiation therapies for their cancer [7]. The capacity of LITT to modulate BBB permeability and activate an immune response via GNS-amplified hyperthermia represents an alluring window of opportunity for the assessment of various combination therapies [1, 2, 8]. Clinical studies of LITT and GNS in companion animal patients seeking treatment for gliomas, such as canines, may further aid in the effective translation of this modality [9].
